# Diaphragm reconstruction by GORE DUALMESH in patients undergoing resection for thoracic malignancies

**DOI:** 10.1186/s13019-021-01449-3

**Published:** 2021-03-31

**Authors:** Atsushi Sano, Yoko Azuma, Takashi Sakai, Satoshi Koezuka, Hajime Otsuka, Akira Iyoda

**Affiliations:** Division of Chest Surgery, Toho University School of Medicine, 6-11-1 Omori-nishi, Ota-ku, Tokyo, 143-8541 Japan

**Keywords:** Diaphragm, Prosthesis, Reconstruction, Lung cancer, Malignant pleural mesothelioma

## Abstract

**Background:**

We used GORE DUALMESH for the reconstruction of diaphragms in patients with thoracic malignancies. Here, we report the results.

**Methods:**

Between July 2015 and August 2017, diaphragm reconstruction using 2-mm GORE DUALMESH was performed in 7 patients undergoing surgical resection for thoracic malignancies. After resection of the diaphragm, the mesh was trimmed to the size of defect and placed with the smooth surface facing the chest cavity and the rough surface facing the abdomen. It was fixed with interrupted sutures consisting of synthetic monofilament nonabsorbable 1–0 to 2 threads.

**Results:**

Indications for resection were malignant pleural mesothelioma and primary lung cancer in 5 and 2 patients, respectively. Patients with malignant pleural mesothelioma underwent pleurectomy with decortication; patients with primary lung cancer underwent lung lobectomy. Right and left diaphragm reconstruction was performed for 4 and 3 patients, respectively. Neither complications related to diaphragm reconstruction nor displacement of mesh occurred during a follow-up period ranging from 11 days to 37 months.

**Conclusions:**

GORE DUALMESH is a good synthetic material for diaphragm reconstruction, because its smooth surface prevents adhesions to the lung and its rough surface allows adherence to abdominal tissue.

## Background

Diaphragm resections are sometimes performed for tumors involving the diaphragm such as malignant pleural mesothelioma and primary lung cancer. Diaphragm reconstruction is performed to avoid respiratory compromise and the displacement of abdominal contents into the chest. Depending on the extent of resection, the diaphragm is sutured only or reconstructed using synthetic material or autologous tissues.^1^^)^ Synthetic material is often used for large defects. Nowadays, polytetrafluoroethylene (PTFE) is the most popular synthetic material, because it provides the necessary strength and is watertight.

GORE® DUALMESH® (W. L. Gore & Associates, Flagstaff, AZ) consists of PTFE sheets with 2 different surfaces.^2^^)^ One surface is smooth and the other is rough. The smooth surface has a microporous surface and is intended not to adhere to the adjacent organ. The rough surface has a macroporous surface and is intended to adhere to the surrounding tissue by allowing in-growth of fibrous tissue. GORE DUALMESH has been successfully used for the repair and reconstruction of the abdominal wall and for procedures correcting a ventral (incisional) hernia or large hiatus hernia.^3–6^^)^ GORE DUALMESH was also recently used for chest wall reconstruction.^7–10^^)^

We used GORE DUALMESH for the reconstruction of the diaphragm. Herein, we report the results of diaphragm reconstruction using GORE DUALMESH for patients undergoing surgical resection for thoracic malignancies.

## Methods

### Patients

This retrospective observational survey was approved by the ethics committee of our institution (No.M19155). Informed consent of patients has been obtained. Between July 2015 and August 2017, diaphragm reconstruction using 2-mm GORE DUALMESH was performed in 7 patients (6 males, 1 female; mean age 70 years, range 62–77 years) (Table 1). Medical records of the patients were reviewed to investigate the clinical characteristics, operative procedure, and postoperative outcomes of the patients.

**Table 1:** Patient details and outcomes

Case	Age	Gender	Diagnosis	Side	Size of mesh after trimming (cm)	Additional procedure	Complication related to reconstruction	Follow-up period
1	76	male	Primary lung cancer, liver invasion	Right	8 × 6	Right lower lobectomy, liver resection	None	13 months
2	73	male	Primary lung cancer, diaphragm invasion	Left	15 × 10	Left lower lobectomy, diaphragm resection	None	24 months
3	74	male	Malignant Pleural mesothelioma	Right	19 × 15	Pleurectomy decortication	None	37 months
4	62	male	Malignant Pleural mesothelioma	Right	19 × 15	Pleurectomy/ decortication	None	14 months
5	64	male	Malignant Pleural mesothelioma	Right	30 × 20	Pleurectomy/ decortication	None	27 months
6	77	male	Malignant Pleural mesothelioma	Left	30 × 20	Pleurectomy/ decortication	None	11 days
7	65	female	Malignant Pleural mesothelioma	Left	30 × 20	Pleurectomy/ decortication	None	18 months

### Surgical technique

The tumor-containing portion of the diaphragm was resected, to include a tumor-free margin with preservation of the peritoneum as far as possible. After resection of the diaphragm, 2-mm GORE DUALMESH was trimmed to accommodate the size of defect. The mesh was placed so that the smooth side faced the chest cavity and the rough side faced the abdominal cavity. The mesh was fixed with interrupted sutures consisting of synthetic monofilament nonabsorbable 1–0 to 2 threads. For partial resection of the diaphragm, the mesh was attached to the diaphragm. For resection of the entire diaphragm, the mesh was attached to the chest wall, usually at the level of the sixth rib to the anterior chest wall and at the level of the ninth rib to the posterior chest wall.

### Three-dimensional computed tomography analysis

Chest computed tomography was performed with 1-mm slice thicknesses without overlap and pitch. Computed tomography images were reconstructed by Horos software, version 4.0 (Horos Project), an open-source medical image viewer.^11, 12^^)^ The data from chest computed tomography scans of patients after they had undergone diaphragm reconstruction with GORE DUALMESH were imported to Horos, and axial views were converted to three-dimensional volume-rendered images. The three-dimensional images were set at window widths of 300 Hounsfield units and window levels of 300 Hounsfield units. The three-dimensional images of GORE DUALMESH were extracted using a trimming function.

## Results

The indications for diaphragm resection included malignant pleural mesothelioma in 5 patients and primary lung cancer in 2 patients. The patents with malignant pleural mesothelioma underwent pleurectomy/decortication, and the patients with primary lung cancer underwent lung lobectomy with concomitant diaphragm resection. One of the primary lung cancer patients underwent concomitant liver resection. Right diaphragm reconstruction was performed for 4 patients and left diaphragm reconstruction was performed for 3 patients. Table 1 summarizes information on the patients and the procedures for each patient.

During follow-up periods ranging between 11 days to 37 months, neither complications related to diaphragm reconstruction nor displacement of mesh occurred. Seromas occurring around the mesh were also not observed.

**Fig. 1:**
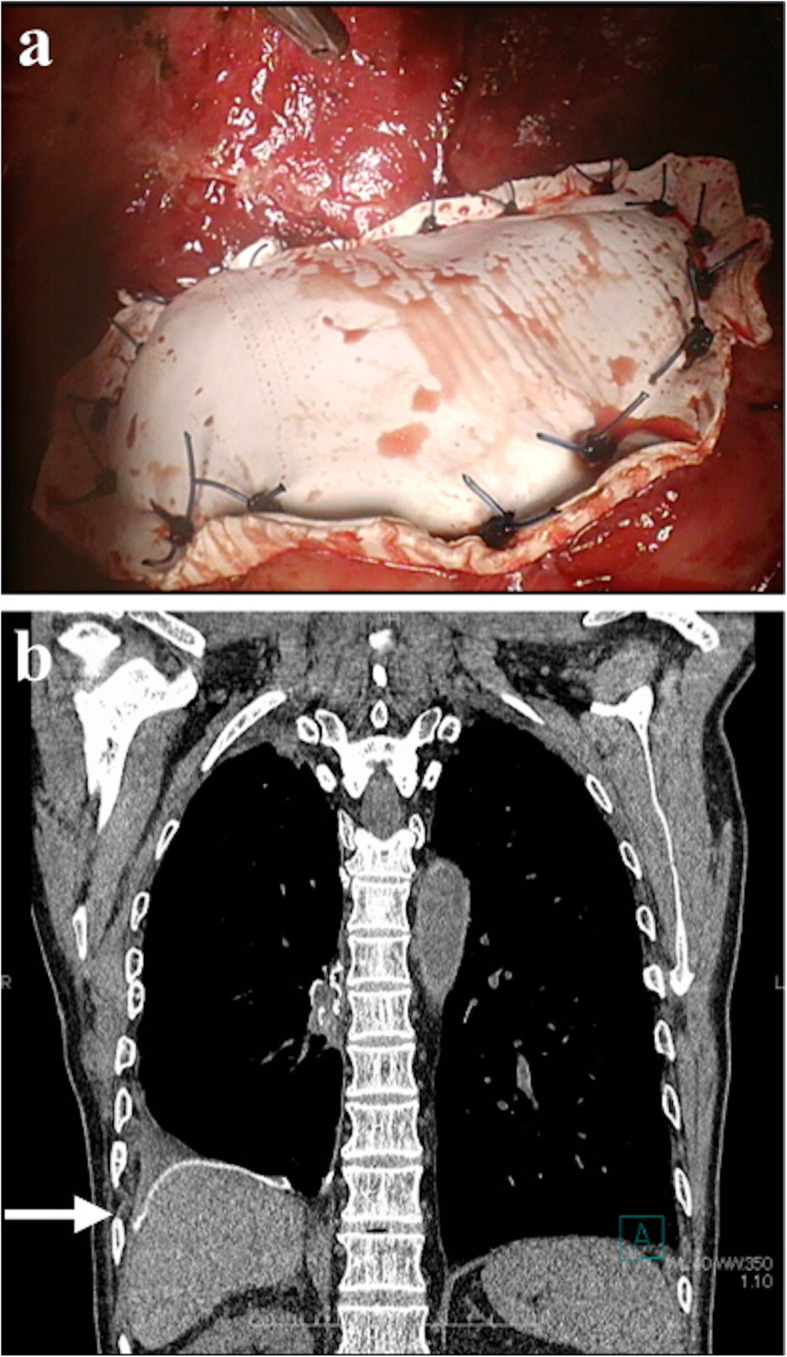
Case 1. A 76-year-old man with primary lung cancer of the right lower lobe invading the right diaphragm and liver. (a) Intraoperative view. Part of the diaphragm was reconstructed with GORE DUALMESH. (b) Computed tomography 1 year after surgery. The mesh remains in place without any sign of complication (white arrow)

Case 1 (Table 1) was a 76-year-old man with primary lung cancer of the right lower lobe that had invaded the right diaphragm and liver. We performed a right lower lobectomy and concomitant resection of diaphragm and liver. The diaphragmatic defect was repaired using GORE DUALMESH (Fig. 1a). The mesh was placed adjacent to the remaining lung and liver. Computed tomography performed 1 year after the operation demonstrated that the mesh remained in place without any evidence of associated complications (Fig. 1b).

**Fig. 2:**
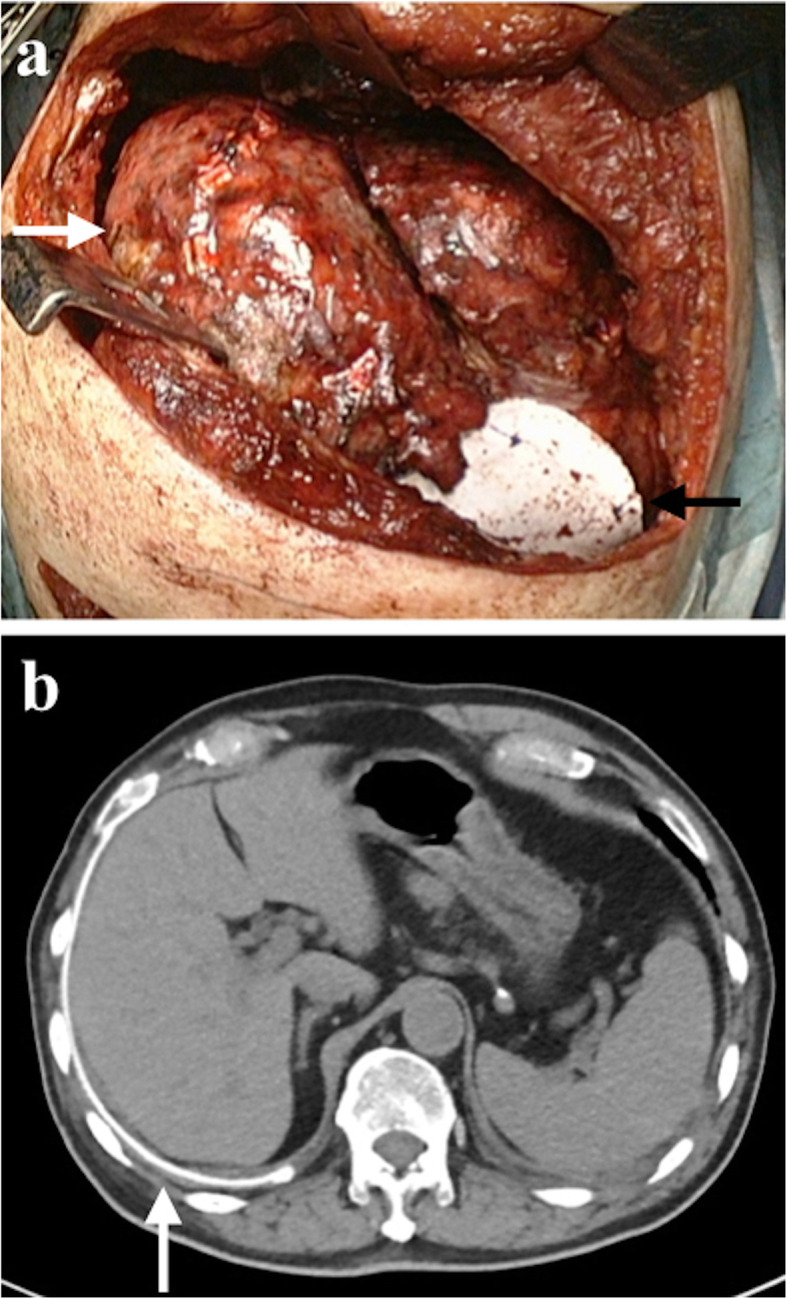
Case 4. A 62-year-old man with a right malignant pleural mesothelioma. (a) Intraoperative view. Right lung after decortication is seen in the left thoracic cavity (white arrow). Part of the right diaphragm was resected, and the defect was repaired with GORE DUALMESH (black arrow). (b) Computed tomography 1 month after surgery. The mesh remains in place without any sign of complication (white arrow)

Case 4 was a 62-year-old man with right malignant pleural mesothelioma. We performed right pleurectomy/decortication. Part of the right diaphragm was resected, and the defect was repaired with GORE DUALMESH (Fig. 2a). The mesh was placed adjacent to lung parenchyma and the peritoneum. Computed tomography performed 1 month after surgery demonstrated that the mesh remained in place without any evidence of associated complications (Fig. 2b).

**Fig. 3:**
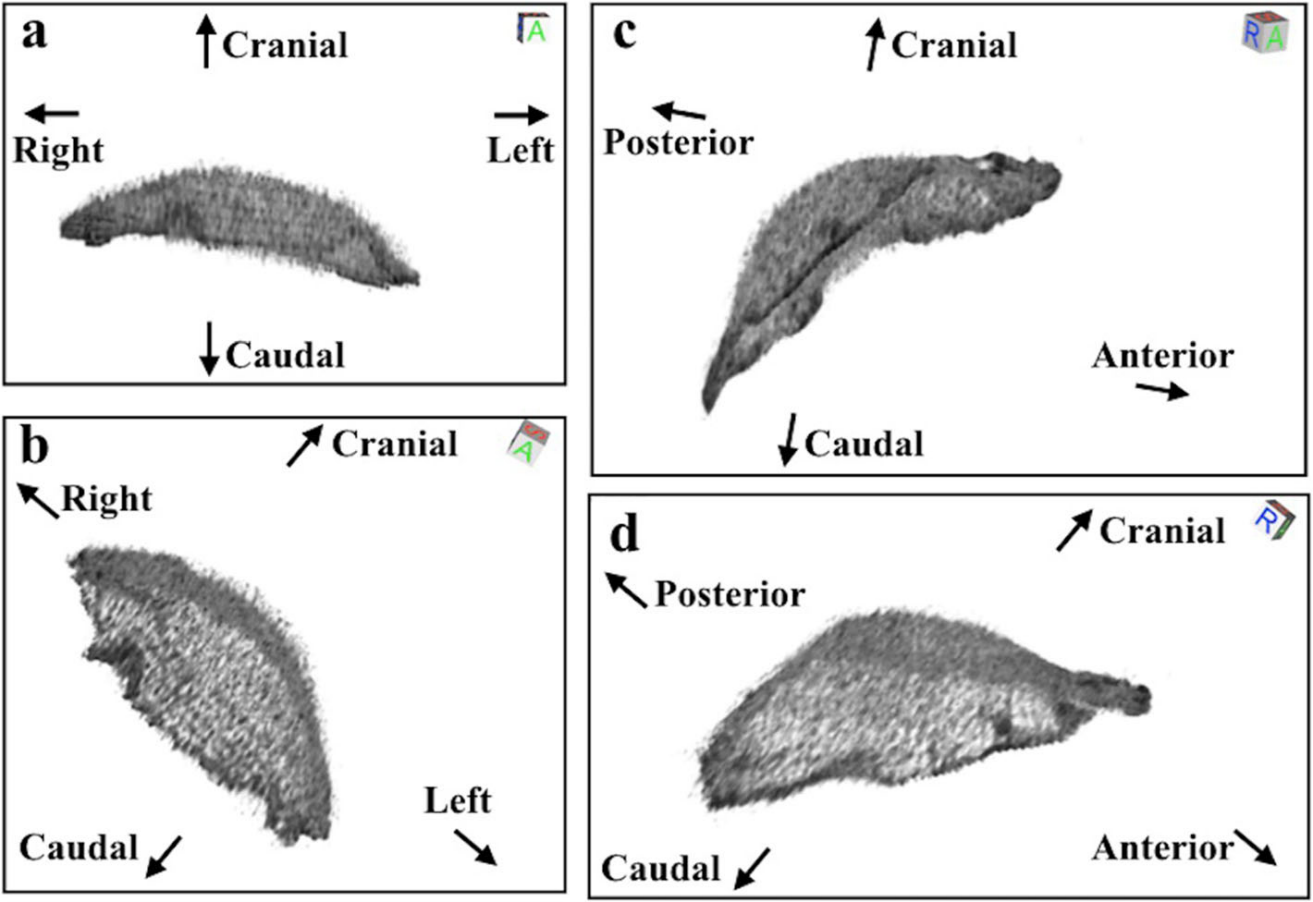
Three-dimensionally rendered postoperative computed tomography images of GORE DUALMESH. (a, b) Case 2. Left posterior portion of diaphragm was resected, and the defect was repaired with GORE DUALMESH in a patient with primary lung cancer with invasion of the diaphragm. The mesh was shaped to accommodate the spleen without distortion. (c, d) Case 3. Right posterior portion of diaphragm was resected, and the defect was repaired with GORE DUALMESH in a patient with malignant pleural mesothelioma. The mesh was shaped to accommodate the liver without distortion

Three-dimensional renderings were performed of available thin-sectional computed tomography images of 2 patients who underwent imaging after repair with GORE DUALMESH. Case 2 was a 73-year-old man with primary lung cancer that had invaded the diaphragm, who underwent left lower lobectomy and concomitant resection of the left diaphragm with repair of the defect. A portion of the left posterior diaphragm was resected, and the defect was repaired with GORE DUALMESH. The mesh was shaped to accommodate the spleen without distortion (Fig. 3a, b). Case 3 was a 74-year-old man who underwent right pleurectomy/decortication for malignant pleural mesothelioma. A portion of the right posterior diaphragm was resected, and the defect was repaired with GORE DUALMESH. The mesh was shaped to accommodate the liver without distortion (Fig. 3c, d).

## Discussion

Diaphragm repair/reconstruction with a synthetic material is sometimes necessary after resection of the diaphragm for malignant disease.^1^^)^ The synthetic materials used for diaphragm reconstruction must be sufficiently strong and durable for a long period of time. PTFE satisfies these requirements, and has been used for diaphragm reconstruction and repair of defects.

GORE DUALMESH is a PTFE sheet with 2 different surfaces.^2^^)^ One surface is a smooth surface and the other surface is a rough surface. The smooth surface is intended not to adhere to the adjacent organ. The rough surface is intended to adhere to the adjacent organ, which prevents the formation of a seroma around the mesh.

GORE DUALMESH has been used for repair of a congenital defect in the abdominal wall and for a ventral hernia (Table 2).^3, 4^^)^ The reported complications related to repair of the abdominal wall include infection, detachment, and seroma. Infections were reported in 4 of 52 cases and 5 of 34 cases. Because GORE DUALMESH is a synthetic material, the prevention of surgical infections is important.

**Table 2:** Previous reports on GORE DUALMESH

Author	Reconstructed organ	Number of cases	Complication related to mesh
Chrysos et al	Abdominal wall (incisional ventral hernia)	52	Wound infection (4 cases), Subcutaneous seroma (8 cases)
Risby et al	Abdominal wall (congenital)	34	Infection (5 cases), detachment (4 cases), suture granulomas (1 case)
Cui et al	Pelvic peritoneum	30	None
Akiba et al	Chest wall	5	Seroma (1 case), chest wall deformity (1 case), pleural effusion (1 case)
Nagayasu et al	Chest wall	11	Paradoxical respirations (1 case)
Our report	Diaphragm	7	None

Subcutaneous seromas which were treated by paracentesis, were reported in 8 of 52 ventral hernia cases.^3^^)^ Although the rough side of GORE DUALMESH is designed to minimize the formation of seromas, they sometime occur.

Reconstruction of the pelvic peritoneum with GORE DUALMESH has also been reported.^6^^)^ The smooth surface of GORE DUALMESH has been found to minimize the formation of intestinal adhesions, and the use of GORE DUALMESH has led to improved bowel movements.

Chest wall reconstructions with GORE DUALMESH have been reported, with complications such as seroma and pleural effusion.^9, 10^^)^ Based on follow-ups of at least 51 months, Nagayasu et al. concluded that GORE DUALMESH has acceptable durability.^10^^)^

In the reconstruction/repair of the diaphragm, preventing adhesion formation between lung and the reconstructed diaphragm is desired for maintenance of the best possible postoperative pulmonary function and for patients that might need additional lung surgery. In this regard, GORE DUALMESH is a good synthetic material for diaphragmatic reconstruction.

None of our patients developed complications related to reconstruction. The most important complication to prevent after repair of the diaphragm is displacement. Securing stable fixation of the mesh is of course essential. In addition, the rough side of a sheet of GORE DUALMESH is intended to adhere to the adjacent organ to prevent the development of seroma, which is thought to contribute to the prevention of mesh displacement. Mesh displacement has been reported to occur late in the postoperative course after chest wall reconstruction. In our case series, the three-dimensional computed tomography images revealed that the mesh was shaped like the original diaphragm and had not been displaced. Therefore, the use of GORE DUALMESH in diaphragm repair is a reasonable option.

In our study, 5 patients with malignant pleural mesothelioma underwent diaphragm resection without opening peritoneum. Two patients with lung cancer underwent diaphragm resection with opening peritoneum. In one patient with right diaphragm resection, the rough surface faced the liver. In the other patient with left diaphragm resection, the rough surface faced to stomach. In both patients, symptoms due to peritoneal adhesion have not occurred. We thought the reason symptoms did not occur is that the area of the resected diaphragm is small. Late complications were not observed in our study patients over a follow-up period of up to 37 months. There is a possibility of late complications including erosion bleeding with the use of synthetic material for the repair of defects.^13^^)^ However, according to previous reports, late complications related to repairs employing GORE DUALMESH are rare. Therefore, GORE DUALMESH is thought to have acceptable durability for diaphragm reconstruction/repair.

The limitations of this study include the small number of cases, a short follow-up period, the retrospective design, and lack of comparison with other materials. To identify the utility and durability of GORE DUALMESH, further long-term follow-up studies and larger numbers of study patients are needed.

## Conclusions

GORE DUALMESH is a good synthetic material for diaphragm reconstruction/repair in patients undergoing surgical resection for thoracic malignancies, not only because of its strength and durability, but also because its smooth surface prevents adhesions to the lung and its rough surface allows adherence to abdominal tissue.

## Data Availability

All data generated or analyzed during this study are included in this published article.

## Abbreviations

PTFE: polytetrafluoroethylene
